# Novel Diagnostic Algorithm for Azoospermia: The Role of 17-Hydroxyprogesterone and Round Spermatids in Normogonadotropic Azoospermia

**DOI:** 10.3390/diagnostics16121830

**Published:** 2026-06-12

**Authors:** Sandro La Vignera, Rosita A. Condorelli

**Affiliations:** Department of Clinical and Experimental Medicine, University of Catania, 95124 Catania, Italy; rosita.condorelli@unict.it

**Keywords:** normogonadotropic azoospermia, 17-hydroxyprogesterone, intratesticular testosterone, round spermatids, hCG therapy, FSH therapy, male infertility, spermatogenesis

## Abstract

Normogonadotropic azoospermia (NOAN) represents a diagnostic and therapeutic challenge in male infertility, affecting men with normal gonadotropin levels but absent sperm in the ejaculate. Emerging evidence has identified 17-hydroxyprogesterone (17OHP) as a potential biomarker for detecting reduced intratesticular testosterone (ITT) levels, and the presence of round spermatids in ejaculate as an indicator of residual spermatogenic activity. This report synthesizes current evidence on a proposed hypothesis-generating diagnostic framework that utilizes these markers to guide hormonal treatment strategies. Specifically, patients with elevated 17OHP levels (>1.18 ng/mL) and detectable round spermatids may benefit from combined human chorionic gonadotropin (hCG) and follicle-stimulating hormone (FSH) therapy at doses lower than those used for hypogonadotropic hypogonadism. However, this cutoff has not been prospectively validated in NOAN-specific cohorts, and the evidence supporting this approach remains preliminary, derived from small heterogeneous cohorts. Alternative therapeutic strategies, including FSH monotherapy and non-hormonal pharmacological treatments, are also discussed. This framework requires rigorous prospective validation before clinical implementation.

## 1. Introduction

Azoospermia, defined as the complete absence of spermatozoa in the ejaculate, affects approximately 1% of all men and 10–15% of infertile men. It is traditionally classified into obstructive azoospermia (OA) and non-obstructive azoospermia (NOA), with the latter representing a heterogeneous group of spermatogenic failures. Within NOA, normogonadotropic azoospermia (NOAN) presents a particularly complex clinical scenario: patients exhibit normal serum follicle-stimulating hormone (FSH) and luteinizing hormone (LH) levels, suggesting intact hypothalamicpituitarygonadal (HPG) axis function, yet fail to produce sperm [1].

The management of NOAN has historically been controversial, with debate surrounding the utility of hormonal interventions in men with apparently normal gonadotropin levels [1,2]. However, emerging evidence suggests that a subset of NOAN patients may have subtle endocrine dysfunction not captured by standard gonadotropin measurements, specifically reduced intratesticular testosterone (ITT) levels despite normal serum testosterone [3,4]. This recognition has prompted the development of novel diagnostic algorithms incorporating biomarkers such as 17-hydroxyprogesterone (17OHP) and the detection of round spermatids in ejaculate to identify patients who may benefit from targeted hormonal therapy [1,5]. It is important to emphasize, however, that multiple therapeutic approaches exist for NOAN, including FSH monotherapy, non-hormonal pharmacological treatments, and empirical hormonal stimulation, each with varying levels of evidence [6,7]. This report presents a preliminary, hypothesis-generating diagnostic framework that requires prospective validation before it can be recommended for routine clinical use. Importantly, this framework is presented as a hypothesis-generating proposal, not as a validated clinical algorithm, and should be interpreted accordingly.

## 2. Background and Theoretical Foundations

### 2.1. Normogonadotropic Azoospermia: Definition and Pathophysiology

NOAN is defined by the presence of azoospermia with serum FSH and LH levels within the normal reference range (typically FSH < 12 IU/L, LH < 10 IU/L).

It should be noted that the FSH cut-off of 12 IU/L for defining normogonadotropic status is not universally recognized across all guidelines and laboratory reference ranges; clinicians should interpret FSH values in the context of local laboratory norms and the overall clinical picture [EAU Guidelines, 2024]. This condition accounts for approximately 10–45% of NOA cases and represents a heterogeneous entity with multiple underlying pathophysiological mechanisms [8,9,10].

### 2.2. The Intratesticular Testosterone Hypothesis

The intratesticular testosterone (ITT) hypothesis posits that adequate local testosterone concentrations within the testicular microenvironment are essential for normal spermatogenesis. Serum testosterone levels may not accurately reflect ITT concentrations, and a subset of NOAN patients may have ITT deficiency despite normal serum testosterone measurements [3,4].

This hypothesis, while biologically plausible, remains to be confirmed through prospective studies with hard clinical endpoints.

## 3. Diagnostic Markers in Normogonadotropic Azoospermia

### 3.1. 17-Hydroxyprogesterone as a Biomarker

17-Hydroxyprogesterone (17OHP) is a steroidogenic intermediate in the androgen synthesis pathway, produced primarily in Leydig cells. Elevated serum 17OHP levels may indicate impaired conversion to androstenedione and testosterone, suggesting a functional block in steroidogenesis [5,11,12,13].

A threshold of 17OHP > 1.18 ng/mL has been proposed as indicative of reduced ITT and potential responsiveness to hCG stimulation [5]. However, it is critical to acknowledge that this cutoff value has not been rigorously validated in prospective NOAN-specific cohorts. The existing evidence supporting this threshold is derived from small, heterogeneous cohorts and indirect measures of ITT. No robust data currently demonstrate that this cutoff reliably predicts hard clinical outcomes in NOAN patients, including: sperm return to the ejaculate after hCG/FSH therapy; surgical sperm retrieval success rates (micro-TESE); or live birth rates following assisted reproduction. The 1.18 ng/mL threshold should therefore be considered exploratory and hypothesis-generating, pending prospective validation in well-defined NOAN cohorts [5,13,14].

### 3.2. Round Spermatids in Ejaculate: Detection and Significance

The presence of round spermatids (immature germ cells) in the ejaculate indicates residual spermatogenic activity arrested at the spermatid stage. Detection of round spermatids using May–Grünwald–Giemsa staining provides a non-invasive indicator of partial spermatogenesis [15,16]. Their presence, when combined with elevated 17OHP, may suggest a potentially reversible form of spermatogenic failure amenable to hormonal stimulation [1,5].

### 3.3. The Proposed Hypothesis-Generating Diagnostic Framework

Based on the available preliminary evidence, a proposed diagnostic framework has been developed to stratify NOAN patients for hormonal therapy. This framework is presented as a hypothesis-generating tool and not as a validated clinical algorithm. Implementation in routine clinical practice should await prospective validation studies with pre-defined hard endpoints.

**Figure 1:**
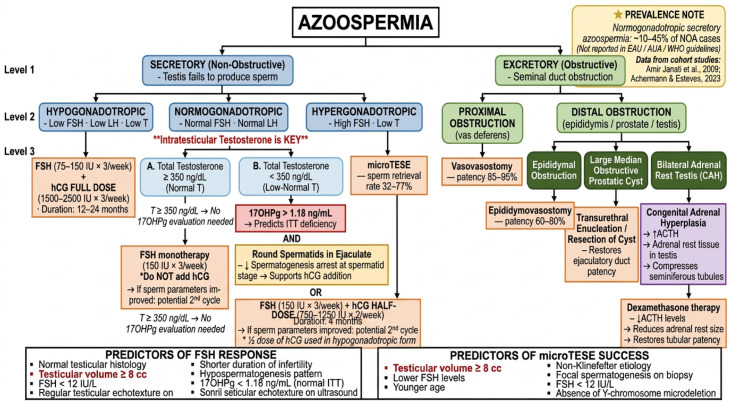
Proposed hypothesis-generating diagnostic framework for azoospermia [1,2,3,6,7,11,13,14,15,17,18,19]. This algorithm requires prospective validation before clinical implementation. Note: low testosterone defined as <300 ng/dL (<10.4 nmol/L) per EAU/AUA guidelines; 350 ng/dL threshold may be functionally low in patients with elevated SHBG. FSH cut-off of 12 IU/L is not universally recognized. * ½ dose of hCG compared to doses used in hypogonadotropic hypogonadism (750–1250 IU × 2/week vs. 1500–2500 IU × 3/week). ** Intratesticular testosterone (ITT) is a key driver of spermatogenesis; its deficiency in normogonadotropic azoospermia is the primary target of the proposed hormonal intervention.

Figure 1 illustrates the proposed diagnostic framework. Key decision points include assessment of total testosterone levels, 17OHP measurement, and detection of round spermatids in ejaculate. Important caveats regarding Figure 1: (1) ‘Low total testosterone’ is defined as <300 ng/dL (<10.4 nmol/L) per EAU/AUA guidelines; however, the threshold of 350 ng/dL shown in the figure may represent functionally low testosterone in patients with elevated sex hormone-binding globulin (SHBG), in whom calculated free testosterone should also be assessed. (2) The FSH cut-off of 12 IU/L for normogonadotropic classification is not universally recognized; local reference ranges and clinical context must be considered.

## 4. Pathophysiological Mechanisms

### 4.1. 17OHP Elevation and Reduced Intratesticular Testosterone

The accumulation of 17OHP in the presence of azoospermia may reflect a functional impairment of the CYP17A1 enzyme (17α-hydroxylase/17,20-lyase), which catalyzes the conversion of 17OHP to androstenedione. This enzymatic dysfunction, even when partial, may result in insufficient ITT despite normal serum testosterone concentrations [11,12].

Abbaticchio et al. (1988) demonstrated that the 17OHP:testosterone ratio and its modification after hCG stimulation could identify men with subtle steroidogenic dysfunction [12].

### 4.2. Steroidogenic Enzyme Dysfunction

Partial CYP17A1 dysfunction represents one of several potential mechanisms underlying ITT deficiency in NOAN. Other contributing factors may include impaired LH receptor sensitivity, altered intratesticular paracrine signaling, and primary Leydig cell dysfunction. The relative contribution of each mechanism likely varies among individual patients, contributing to the heterogeneity of treatment responses observed in clinical studies [3,4,11,20].

## 5. Treatment Strategies

### 5.1. Rationale for Hormonal Therapy in Normogonadotropic Azoospermia

The theoretical rationale for hormonal stimulation in NOAN patients with ITT deficiency is based on the premise that exogenous gonadotropin administration can augment ITT concentrations above the threshold required for spermatogenesis. This approach has been validated in hypogonadotropic hypogonadism, where combined hCG/FSH therapy reliably induces spermatogenesis [17,18,20,21,22].

### 5.2. Combined hCG and FSH Therapy

In NOAN patients with 17OHP > 1.18 ng/mL and detectable round spermatids, combined hCG and FSH therapy at reduced doses (approximately half of those used in hypogonadotropic hypogonadism) has been proposed as a therapeutic strategy. The rationale for dose reduction is that the HPG axis is partially functional in NOAN, requiring less exogenous stimulation than in complete gonadotropin deficiency [1,4,23,24,25].

Gialouris et al. (2025) recently demonstrated the efficacy of gonadotropin therapy in inducing spermatogenesis in men with pathologic gonadotropin deficiency, providing updated evidence on dosing protocols and outcomes [10].

### 5.3. Alternative Therapeutic Approaches

It is essential to acknowledge that alternative therapeutic approaches exist for NOAN and idiopathic male infertility, supported by varying levels of evidence:

- FSH Monotherapy: Zhang et al. (2022) demonstrated in a systematic review and meta-analysis that FSH therapy can improve semen parameters in patients with idiopathic oligoasthenoteratozoospermia, suggesting FSH monotherapy as a viable alternative in selected patients [26].
- Non-Hormonal Pharmacological Treatments: Al Wattar et al. (2024) conducted a systematic review and network meta-analysis of non-hormonal pharmacological options for male infertility, including clomiphene citrate, tamoxifen, aromatase inhibitors, and antioxidants [27]. Clomiphene showed the highest likelihood of improving sperm concentration, though evidence quality was low and no treatment showed clear superiority over placebo for live birth rates [27].

Empirical FSH + hCG: Combined therapy remains an option in selected cases, but its superiority over monotherapy has not been established in prospective RCTs in NOAN.

### 5.4. Dosing Protocols: Half-Dose Strategy

The proposed half-dose strategy for NOAN involves administering hCG at 750–1250 IU twice weekly (compared to 1500–2500 IU three times weekly in hypogonadotropic hypogonadism) in combination with FSH at 75–150 IU three times weekly. Treatment duration of at least 4 months is recommended before assessing spermatogenic response, consistent with the duration of a complete spermatogenic cycle [1,4].

### 5.5. Treatment Algorithm Based on 17OHP and Round Spermatids

The proposed treatment pathway stratifies NOAN patients as follows: patients with total testosterone ≥350 ng/dL and 17OHP ≤ 1.18 ng/mL are candidates for FSH monotherapy; patients with total testosterone <350 ng/dL or 17OHP > 1.18 ng/mL with round spermatids are candidates for combined hCG + FSH half-dose therapy. This stratification is proposed as a hypothesis-generating framework and must be validated in prospective studies before adoption in clinical guidelines. In patients with borderline testosterone (300–350 ng/dL) and elevated SHBG, assessment of calculated free testosterone is recommended to avoid misclassification.

## 6. Clinical Outcomes and Evidence

### 6.1. Effects on Intratesticular Testosterone

Studies examining the effects of hCG therapy on ITT in NOAN patients have reported increases in serum testosterone and 17OHP normalization in responders. However, these studies are limited by small sample sizes, retrospective designs, and lack of direct ITT measurement (which requires testicular biopsy or fine-needle aspiration) [3,4,23,24].

### 6.2. Sperm Retrieval and Spermatogenesis Outcomes

Available data on sperm retrieval outcomes following hCG/FSH therapy in NOAN patients are limited to small case series and retrospective cohorts. Reported rates of sperm appearance in ejaculate range from 20–40% in selected patients, but these figures should be interpreted with caution given the absence of controlled prospective studies [1,23,28,29,30,31].

No prospective randomized controlled trial has specifically evaluated the 17OHP-guided treatment algorithm in NOAN patients with pre-defined endpoints of sperm retrieval success or live birth rates. This represents a critical evidence gap.

### 6.3. Pregnancy and Live Birth Rates

Data on pregnancy and live birth rates following hormonal stimulation in NOAN are extremely limited. Available reports are based on small series without appropriate control groups. The relationship between 17OHP normalization, sperm retrieval, and ultimately live birth has not been established [1,23].

## 7. Discussion

### 7.1. Clinical Implications

The proposed diagnostic framework offers a biologically plausible approach to identifying a subset of NOAN patients who may benefit from hormonal stimulation. The use of 17OHP as a proxy for ITT deficiency provides a non-invasive, clinically accessible biomarker that could complement standard hormonal evaluation. However, clinicians should be aware that this framework has not been validated and should not replace individualized clinical judgment or established guidelines. Alternative approaches—including FSH monotherapy and non-hormonal treatments—should be considered and discussed with patients [6,7].

### 7.2. Limitations and Gaps in Current Evidence

- The proposed diagnostic algorithm is hypothesis-generating and has not been prospectively validated in NOAN-specific cohorts.
- The 17OHP cutoff of 1.18 ng/mL was derived from limited, heterogeneous datasets and has not been validated to predict hard clinical outcomes (sperm retrieval, live birth).
- Supporting evidence relies heavily on indirect measures of ITT and small retrospective cohorts.
- No prospective randomized controlled trial has tested the 17OHP-guided treatment algorithm in NOAN with pre-defined primary endpoints.
- The FSH cut-off of 12 IU/L for normogonadotropic classification is not universally recognized across international guidelines.
- The testosterone threshold of 350 ng/dL may be functionally low in patients with elevated SHBG; free testosterone assessment is recommended in borderline cases.
- Many cited studies were conducted over a decade ago; more recent high-quality evidence is needed to confirm or refute the proposed approach.
- Alternative therapeutic approaches (FSH monotherapy, clomiphene, non-hormonal treatments) have not been systematically compared to the proposed algorithm.
- Technical variability in 17OHP assay methods across laboratories may affect the reproducibility of the proposed cutoff.
- Long-term outcomes, safety data, and cost-effectiveness of the proposed treatment strategy have not been evaluated.

## 8. Future Directions and Recommendations

To advance from a hypothesis-generating framework to a validated clinical tool, the following research priorities are proposed:

1. Prospective multicenter RCTs in well-defined NOAN cohorts comparing 17OHP-guided hCG/FSH therapy versus FSH monotherapy versus watchful waiting, with primary endpoints of sperm return to ejaculate and live birth rates.
2. Standardization of 17OHP assay methodology and establishment of laboratory-specific reference ranges to ensure reproducibility of the proposed cutoff.
3. Direct ITT measurement studies to validate 17OHP as a reliable proxy for ITT deficiency in NOAN patients.
4. Head-to-head comparison of the proposed algorithm with alternative approaches (FSH monotherapy, clomiphene citrate, non-hormonal treatments) to establish relative efficacy.
5. Development of composite predictive models incorporating 17OHP, testosterone, SHBG, testicular volume, and genetic factors to improve patient stratification.

## 9. Conclusions

NOAN remains a complex diagnostic and therapeutic challenge. The use of 17OHP and round spermatids as stratification markers represents a biologically plausible hypothesis for identifying a subset of patients who may benefit from targeted hormonal stimulation. However, this diagnostic and therapeutic framework must be explicitly understood as hypothesis-generating rather than a validated clinical algorithm. The 17OHP cutoff of 1.18 ng/mL lacks prospective validation in NOAN-specific cohorts for hard clinical outcomes. Multiple alternative therapeutic strategies exist and should be considered. Rigorous prospective randomized controlled trials with pre-defined hard endpoints—including sperm retrieval success, pregnancy rates, and live birth rates—are urgently needed before this framework can be recommended for routine clinical implementation.

## Data Availability

No new data were created or analyzed in this study. Data sharing is not applicable to this article.
